# Supplementary material for: Iterative improvement in the automatic modular design of robot swarms
Source: PeerJ Comput Sci. 2020 Dec 7;6:e322. doi: 10.7717/peerj-cs.322 (PMC7924708; doi:10.7717/peerj-cs.322)
Supplement: Supplemental Information 3 [file peerj-cs-06-322-s003.zip › argos3/doc/api/standalone/a00585_source.html]

ARGoS: plugins/simulator/entities/proximity\_sensor\_equipped\_entity.h Source File


- Main Page
- Related Pages
- Namespaces
- Classes
- Files

- File List
- File Members

# plugins/simulator/entities/proximity\_sensor\_equipped\_entity.h

Go to the documentation of this file.

```
00001 
00007 #ifndef PROXIMITY_SENSOR_EQUIPPED_ENTITY_H
00008 #define PROXIMITY_SENSOR_EQUIPPED_ENTITY_H
00009 
00010 namespace argos {
00011    class CProximitySensorEquippedEntity;
00012 }
00013 
00014 #include <argos3/core/utility/math/vector3.h>
00015 #include <argos3/core/simulator/entity/entity.h>
00016 #include <argos3/core/simulator/entity/embodied_entity.h>
00017 #include <map>
00018 
00019 namespace argos {
00020 
00021    class CProximitySensorEquippedEntity : public CEntity {
00022 
00023    public:
00024 
00025       ENABLE_VTABLE();
00026 
00027       struct SSensor {
00028          typedef std::vector<SSensor*> TList;
00029 
00030          CVector3 Offset;
00031          CVector3 Direction;
00032          SAnchor& Anchor;
00033 
00034          SSensor(const CVector3& c_offset,
00035                  const CVector3& c_direction,
00036                  Real f_range,
00037                  SAnchor& s_anchor);
00038       };
00039 
00040    public:
00041 
00042       CProximitySensorEquippedEntity(CComposableEntity* pc_parent);
00043 
00044       CProximitySensorEquippedEntity(CComposableEntity* pc_parent,
00045                                      const std::string& str_id);
00046 
00047       virtual ~CProximitySensorEquippedEntity();
00048 
00049       virtual void Init(TConfigurationNode& t_tree);
00050 
00051       virtual std::string GetTypeDescription() const {
00052          return "proximity_sensors";
00053       }
00054 
00055       virtual void Enable();
00056 
00057       virtual void Disable();
00058 
00059       inline size_t GetNumSensors() const {
00060          return m_tSensors.size();
00061       }
00062 
00063       inline SSensor& GetSensor(size_t un_idx) {
00064          return *m_tSensors[un_idx];
00065       }
00066 
00067       inline SSensor::TList& GetSensors() {
00068          return m_tSensors;
00069       }
00070 
00071       void AddSensor(const CVector3& c_offset,
00072                      const CVector3& c_direction,
00073                      Real f_range,
00074                      SAnchor& s_anchor);
00075 
00076       void AddSensorRing(const CVector3& c_center,
00077                          Real f_radius,
00078                          const CRadians& c_start_angle,
00079                          Real f_range,
00080                          UInt32 un_num_sensors,
00081                          SAnchor& s_anchor);
00082 
00083       void AddSensorFan(const CVector3& c_center,
00084                         Real f_radius,
00085                         const CRadians& c_start_angle,
00086                         const CRadians& c_end_angle,
00087                         Real f_range,
00088                         UInt32 un_num_sensors,
00089                         SAnchor& s_anchor);
00090 
00091    protected:
00092 
00094       SSensor::TList m_tSensors;
00095 
00096    };
00097 
00098 }
00099 
00100 #endif
```

---

Generated on 10 Jul 2018 for ARGoS by 
 1.6.1
